# Polypropylene wiping cloths contaminated with *S. aureus* and *P. aeruginosa* transfer significantly less to uncontaminated surfaces compared to microfiber and cotton

**DOI:** 10.1017/ash.2026.10360

**Published:** 2026-05-18

**Authors:** Maxwell Voorn, Geraldine Tembo, Siddharth Kumar, Haley Oliver-Jischke, Peter Teska

**Affiliations:** 1 Purdue University, USA; 2 Diversey - a Solenis Company of Fort Mill, USA

## Abstract

**Background:**

Cross-contamination from contaminated wiping cloths is an underrecognized route of pathogen transmission in healthcare settings. Reused, misapplied, or ineffective disinfectants can transfer viable bacteria between surfaces. *Staphylococcus aureus* and *Pseudomonas aeruginosa* persist under hospital conditions and are frequently isolated in healthcare environments. While many studies assess bacterial inactivation, few examine how chemistry and wipe material influence transfer when bacterial inactivation is incomplete.

**Methods:**

Hydrogen peroxide (HP), a quaternary ammonium compound (QAC), and an ethoxylated alcohol (EA) cleaner were tested with cotton, microfiber, and meltblown polypropylene wipes. Wipes inoculated with ∼ 7 log_10_ CFU of *S. aureus* or *P. aeruginosa* were used on 1 ft^2^ Formica. Bacterial transfer to surfaces and gloves was quantified between the chemistry and material combinations using single- and multi-layer setups.

**Results:**

HP-based wipes demonstrated the highest bactericidal efficacy, transferring little to no viable bacteria. Polypropylene wipes consistently transferred fewer bacteria than cotton or microfiber wipes. Wipes with QAC or EA, particularly those made of cotton, transferred significantly more bacteria to surfaces and gloves. Adding layers of wipe material reduced bacterial transfer, especially for microfiber.

**Conclusion:**

Chemistry and wipe material both significantly impact bacterial transfer during cleaning. HP with polypropylene wipes offered the most effective reduction in cross-contamination. Cotton and microfiber wipes paired with QAC or EA exhibited higher transfer risks, likely due to electrostatic interactions contributing to reduced antimicrobial activity. These findings emphasize the need for optimized wipe-chemistry combinations to ensure effective surface decontamination.

## Introduction

Healthcare-acquired infections from *Staphylococcus aureus* and *Pseudomonas aeruginosa* have resulted from hospital surface cross-contamination, emphasizing the need for enhanced disinfection methods.^1,2^ Ready-to-use disinfectants and wiping cloths are commonly used for disinfection of hospital and healthcare surfaces.^3^ Wipes impregnated with disinfectant and sanitizing liquids and applied with disposable nitrile or latex gloves are one of the most common methods for decontamination.^4,5^ The widespread use of disposable gloves reduces bacterial contamination compared to bare hands.^6^ However, prolonged glove use allows bacterial accumulation and elevates the risk of cross-contamination.^7–9^ Additionally, wipe substrate material can interact with disinfectant active ingredients, affecting their availability at the point of contact.^10,11^

Prior studies have evaluated disinfectant efficacy and the elimination of *S. aureus* and *P. aeruginosa* ^12–14^ However, few studies have examined cross-contamination when bacteria are incompletely inactivated.^15^ We hypothesized that strong antimicrobial agents, such as hydrogen peroxide (HP), would lead to lower amounts of bacterial transfer compared to quaternary ammonium compound (QAC) based sanitizers and ethoxylated alcohol (EA) based neutral cleaners. We also hypothesized that cotton and microfiber wipes may show greater cross-contamination risks when paired with QACs due to binding of the negatively charged anionic groups found on the surface of the wipe material.^16^ The objectives of this study were to (i) evaluate the risk of *S. aureus* and *P. aeruginosa* transfer from an inoculated multi-layered disposable cloth to a sterile disposable glove and uncontaminated hard non-porous surface, and (ii) determine the optimal chemistry (QAC, EA, or HP) and wipe (cotton, microfiber, or polypropylene) combination that can minimize the transfer of bacteria during cleaning and disinfection.

## Materials and methods

### Surface type, bacterial strains, active ingredients, and material types used in study

Bactericidal efficacy of EPA-registered disinfectants, sanitizers and cleaners (Table 1) was tested in combination with three wiping materials (Table 2) against *P. aeruginosa* (ATCC 15,442) and *S. aureus* (ATCC 6,538). Each combination of chemistry and material type were tested at label contact times. Three ingredient types were evaluated: EA, HP and quaternary ammonium (QAC).

**Table 1.** Active ingredients, dilution for use, and label contact time of disinfectant products used in the study

Acronym	Liquid product active ingredient(s)^a^	Dilution for use	Label contact time (min)^b^
HP^c^	0.5% Hydrogen Peroxide	RTU	1 min
EA^d^	7–13% Ethoxylated Alcohol + 10–30% Ethoxylated Secondary Alcohol	1:256	10 min
QAC2^c^	5.0% Alkyl dimethyl benzyl ammonium chloride + 5.0% Alkyl dimethyl ethylbenzyl ammonium chloride	1:512	3 min

a Active ingredients concentration.

b Defined label contact time for standard use against *S. aureus* and *P. aeruginosa.*

c EPA claim at disinfectant concentration against *S. aureus* (List H).

d No EPA claim against *S. aureus* or *P. aeruginosa.*

**Table 2.** Wipe, material, and dimensions used in the study

Wipe	Fiber/polymer composition	Fabric construction	Dry weight	Dimensions
Cotton^a^	100% Cellulose	Spun terry-weave	62.18 ± 2.52 g	14.5” x 14.5” 36.83 cm x 36.83 cm
Microfiber^a^	90% Polyester and 10% polyamide	High temperature extrusion and knitted split-fiber	28.45 ± 0.03 g	16” x 12” 40.64 cm x 30.48 cm
Polypropylene^b^	100% Polypropylene	Meltblown nonwoven	2.84 ± 0.03 g	12” x 10.5” 30.55 cm x 26.667 cm

a Distributed by Amazon Basics.

b Distributed by Diversey Holdings.

**Figure 1.**
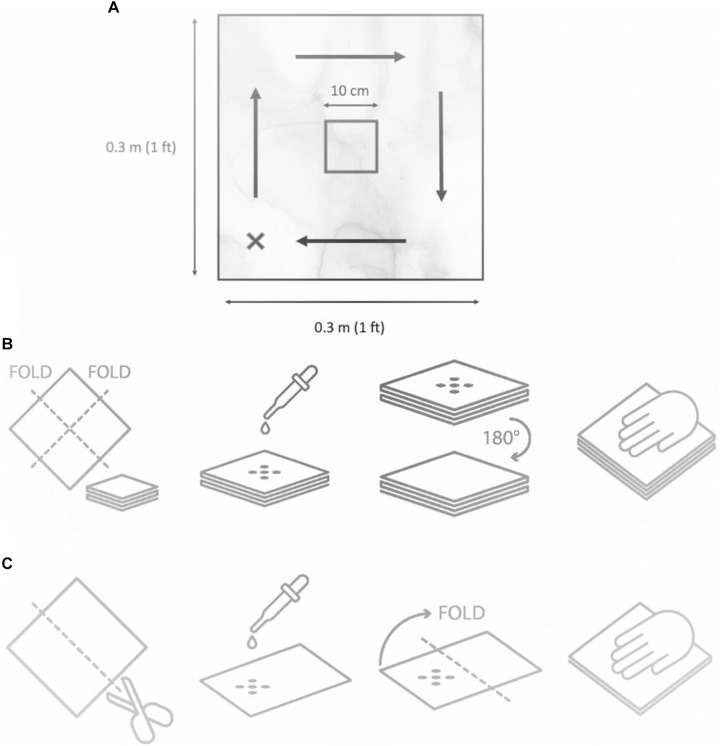
Panel **A**. Formica surface schematic for disinfectant towelette efficacy testing against *Pseudomonas aeruginosa* (ATCC 15,442) and *Staphylococcus aureus* (ATCC 6,538). Inocula were spot-applied to the wipe (black dots); recovery was from the marked square after wiping. Panel **B**. Cloth preparation for evaluation 1: folded twice (four layers), bottom layer inoculated with five 10-µL aliquots of test stock. Panel **C**. Cloth preparation for evaluation 2: cloth cut in half and folded once (two layers), with five 10-µL aliquots applied to the interior side of the top layer before folding.

To ensure uniform liquid permeation throughout the wiping material, the solution was added to the wipe before cleaning. Liquid volumes were determined by each material’s absorptive capacity to achieve full saturation without excess dripping, consistent with standard practice for disinfectant wipe testing and as previously described in Voorn et al.^10,17,18^ The three wipes differ in dry weight, fiber structure, and absorbent capacity, consistent with the typical size range available for each material (Table 2). Cotton terry-weave cloths (62.18 ± 2.52 g) and microfiber knitted split-fiber cloths (28.45 ± 0.03 g) are hydrophilic with high liquid retention, while meltblown polypropylene nonwoven wipes (2.84 ± 0.03 g) are hydrophobic with a much lower absorbent capacity.^11,19^ Applying identical volumes across materials would result in over-saturation of polypropylene or insufficient wetting of cotton and microfiber. For the first evaluation, 15 mL of the liquid product was applied to the meltblown polypropylene (Diversey Holdings Ltd., Fort Mill, SC), 146 mL was applied to cotton cloths (Amazon Inc., Seattle, WA) and 121 mL was applied to microfiber cloths (Amazon Inc., Seattle, WA). For the second evaluation, each wiping material was cut in half, and half the amount of liquid product was used compared to the first evaluation. For this evaluation, 7.5 mL of the liquid product was applied to the dry EasyWipe (Diversey Holdings Ltd., Fort Mill, SC), 73 mL was applied to cotton cloths (Amazon Inc., Seattle, WA) and 60.5 mL was applied to microfiber cloths (Amazon Inc., Seattle, WA) to ensure consistent liquid amount contained in the wipes for the first and second evaluation. The test surface was a 1 ft x 1 ft imitation-granite surface made of laminated Formica commonly used in healthcare facilities represented in Figure 1A.

### Disinfection of the test surface, inoculation, and wiping method

The Formica surface was disinfected and prepared as described in Voorn et al.^18^ *S. aureus* and *P. aeruginosa* inoculum were prepared following EPA MLB SOP-MB-09–08 to test the efficacy of disinfectant towelettes.^20^ A soil load test suspension of 500 µL consisted of 25 µL of 5% bovine serum albumin (BSA; Fisher Bioreagents, Ottawa, Canada), 35 µL of 5% yeast extract (ACROS Organics, New Jersey, US), 100 µL of 0.4% mucin stock (Abnova, Walnut, USA), and 340 µL of bacterial inoculum.

Wipes were sterilized in 70% ethanol for 10 min, then air-dried for at least 1 h before liquid chemistry was applied. In the first experiment, wipes were folded into four layers with the bottom layer inoculated (Figure 1B). In the second experiment, wipes were cut in half, inoculated on the interior surface, and folded once so that there was an uninoculated layer of cloth beneath (Figure 1C). In both scenarios, the gloved hand contacted the backside of the inoculated cloth while wiping the Formica board.

### Bactericidal efficacy testing from the Formica surface and glove

The bacterial suspensions combined with the soil load were inoculated directly into the wipe as depicted by black dots in the schematic diagram (Figure 1A). Five 10 µl aliquots of the test suspension were inoculated onto the surface of the wipe, totaling ∼ 7 log_10_ CFU. The i-zone was comprised of a 10x10 cm square in the center of the Formica sheet. The wiping procedure was done in a circular motion on the Formica surface, completing four revolutions to ensure thorough coverage of the board. After wiping, products were left for the label contact time, then sampling zones were swabbed with PUR-Blue swabs in 10 mL HiCap neutralizing buffer (World Bioproducts, Libertyville, IL). The glove was then placed in a sterile Whirlpak bag with 50 mL neutralizing buffer for stomaching. Samples were serially diluted in PBS, filtered (0.22 µm), and plated on Tryptic Soy Agar. Each condition was tested in triplicate with three biological replicates.

### Statistical analyses

SAS v. 9.4 (SAS Institute, Cary, NC) was used to perform all statistical analyses. The CFU collected from the sample areas were log_10_ transformed and normalized against control log_10_ densities to calculate log_10_ reductions. All bactericidal efficacy data were transformed into log_10_ reduction values for analyses to maximize fit. The analyses had a defined *α* = 0.05 significance level. To determine the factors significantly impacting bacterial reduction, data were fitted to a linear mixed model to analyze the significant differences between material types tested. Both the first and second test for this study evaluated 324 observations for a combined total (N = 648).

## Results

### HP disinfectant demonstrated greater bactericidal efficacy than EA cleaner and QAC sanitizer

**Figure 2.**
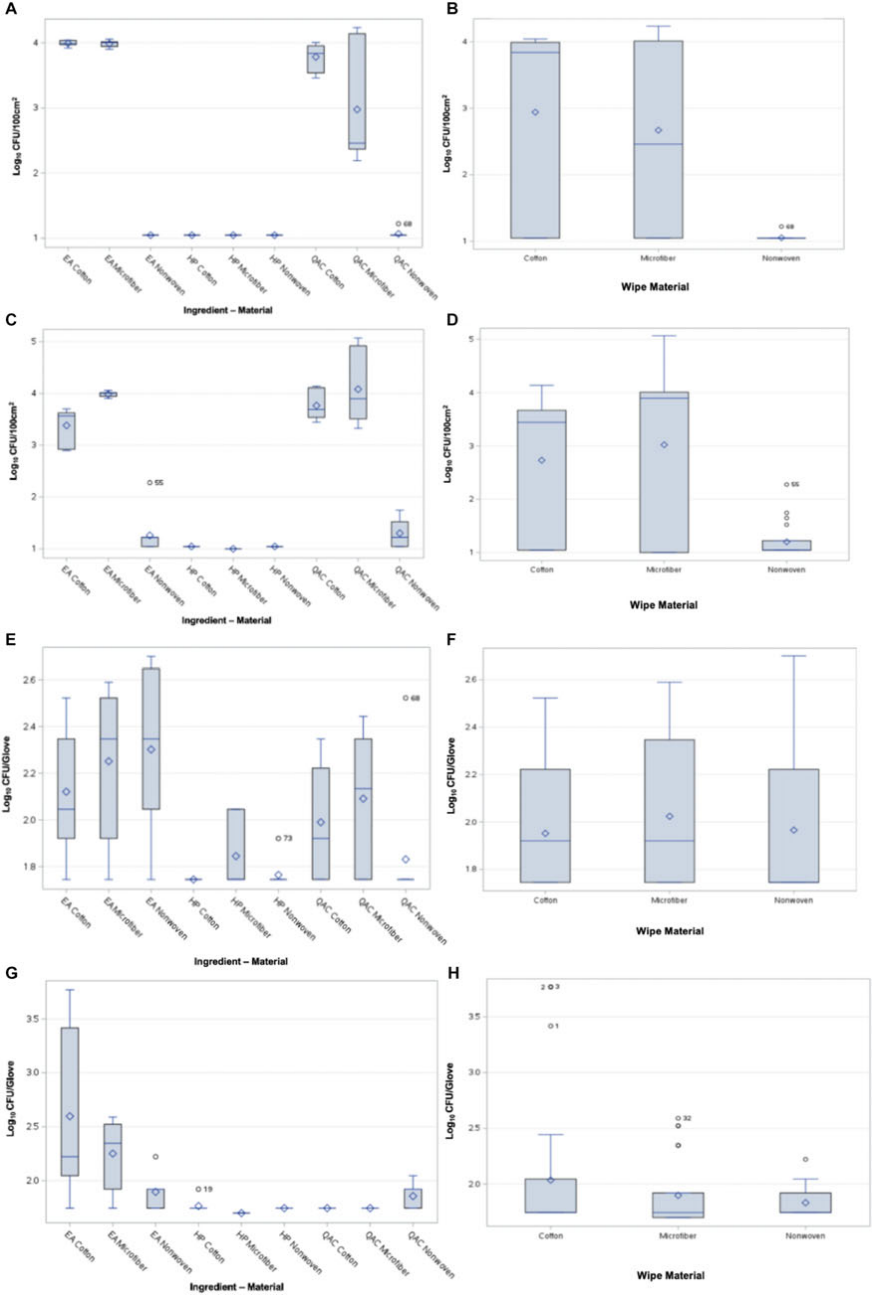
Panel **A** and **B**. Comparison of *S. aureus* transferred from inoculated wipe to the board for each active ingredient and material type combination. The inoculated surface of the wiping cloth was placed directly on the Formica board. Panel **C** and **D**. Comparison of viable *P. aeruginosa* transferred from the wipe to the board for each active ingredient and material type combination. Panel **E** and **F**. Comparison of viable *S. aureus* transferred from the wipe to the glove for each active ingredient and material type combination. Four layers of wiping material were present between the inoculated wipe and glove surface. Panel **G** and **H**. Comparison of viable *P. aeruginosa* transferred from the wipe to the glove for each active ingredient and material type combination. Four layers of wiping material were present between the inoculated wipe and glove surface.

Across all tests, HP-based disinfectants showed strong bactericidal efficacy, leaving bacteria at or below the detection limit on boards and gloves (Figures 2A,C). HP wipes transferred significantly fewer bacteria than both QAC-based sanitizer and EA-based neutral cleaner liquid formulations for both organisms (*P*<.05; Figures 2B,D). Wipes inoculated with ∼ 7 log_10_ CFU of *S. aureus* transferred ≤1.0 ± 0.1 log_10_ CFU/100 cm^2^ to uncontaminated Formica surfaces using the HP-based disinfectant. In contrast, QAC-impregnated wipes transferred 2.6 ± 1.3 log_10_ CFU/100 cm^2^ and EA-impregnated wipes transferred 3.0 ± 1.4 log_10_ CFU/100 cm.^2^

For wipes inoculated with *P. aeruginosa*, ≤1.0 ± 0.1 log_10_ CFU/100 cm^2^ was transferred using HP-based disinfectants. QAC-impregnated wipes transferred 3.0 ± 1.3 log_10_ CFU/100 cm,^2^ while EA-impregnated wipes transferred 2.9 ± 1.2 log_10_ CFU/100 cm^2^ to uncontaminated surfaces, both significantly greater than HP (*P*<.05; Figure 2D).

### Wiping material significantly affected surface cross-contamination risk

The risk of cross-contamination to uncontaminated Formica surfaces was significantly influenced by the combination of wiping material and active liquid ingredient for both *P. aeruginosa* and *S. aureus* (*P*<.0001 and *P*<.0001, respectively; Figures 2A,D). Polypropylene wipes placed directly on Formica transferred significantly fewer bacteria than cotton or microfiber wipes (*P*<.05; Figures 2B,D).

For *S. aureus*, cotton wipes transferred 2.9 ± 1.4 log_10_ CFU/100 cm^2^ and microfiber wipes transferred 2.7 ± 1.3 log_10_ CFU/100 cm,^2^ compared to 1.1 ± 0.1 log_10_ CFU/100 cm^2^ from polypropylene wipes (*P*<.05; Figure 2B). For *P. aeruginosa*, cotton and microfiber wipes transferred 2.7 ± 1.3 and 3.0 ± 1.5 log_10_ CFU/100 cm,^2^ respectively, while polypropylene wipes transferred 1.2 ± 0.3 log_10_ CFU/100 cm^2^ (*P*<.05; Figure 2D).

### Four wipe layers reduced bacterial transfer to gloves

Low levels of bacterial transfer were observed through four layers of wiping material to gloves. The limit of detection was 1.7 log_10_ CFU/glove for glove samples and 1.1 log_10_ CFU/100 cm^2^ for surface samples. Most wipes transferred<2.5 log_10_ CFU/glove regardless of chemistry (Figures 2F,H). No significant differences among wiping materials were observed for *S. aureus* glove transfer (*P* = .5086; Figure 2F).

Significant differences were observed among liquid active ingredients for *P. aeruginosa* glove transfer (*P*<.0001; Figure 2G). The interaction between wiping material and liquid active ingredient was not significant for *S. aureus* (*P* = .3365; Figure 2E) but was significant for *P. aeruginosa* (*P* = .0016; Figure 2G), with material type alone also contributing modestly (*P* = .0449; Figure 2G).

For *S. aureus*, microfiber, cotton, and polypropylene wipes each transferred 2.0 ± 0.3 log_10_ CFU/glove, with no significant differences observed (*P*>.05; Figure 2F). For *P. aeruginosa*, cotton wipes transferred 2.0 ± 0.6 log_10_ CFU/glove, while microfiber and polypropylene wipes transferred 1.9 ± 0.3 and 1.8 ± 0.1 log_10_ CFU/glove, respectively, with significant differences observed (*P*<.05; Figure 2H).

### Addition of a wipe layer impacted cross-contamination

**Figure 3.**
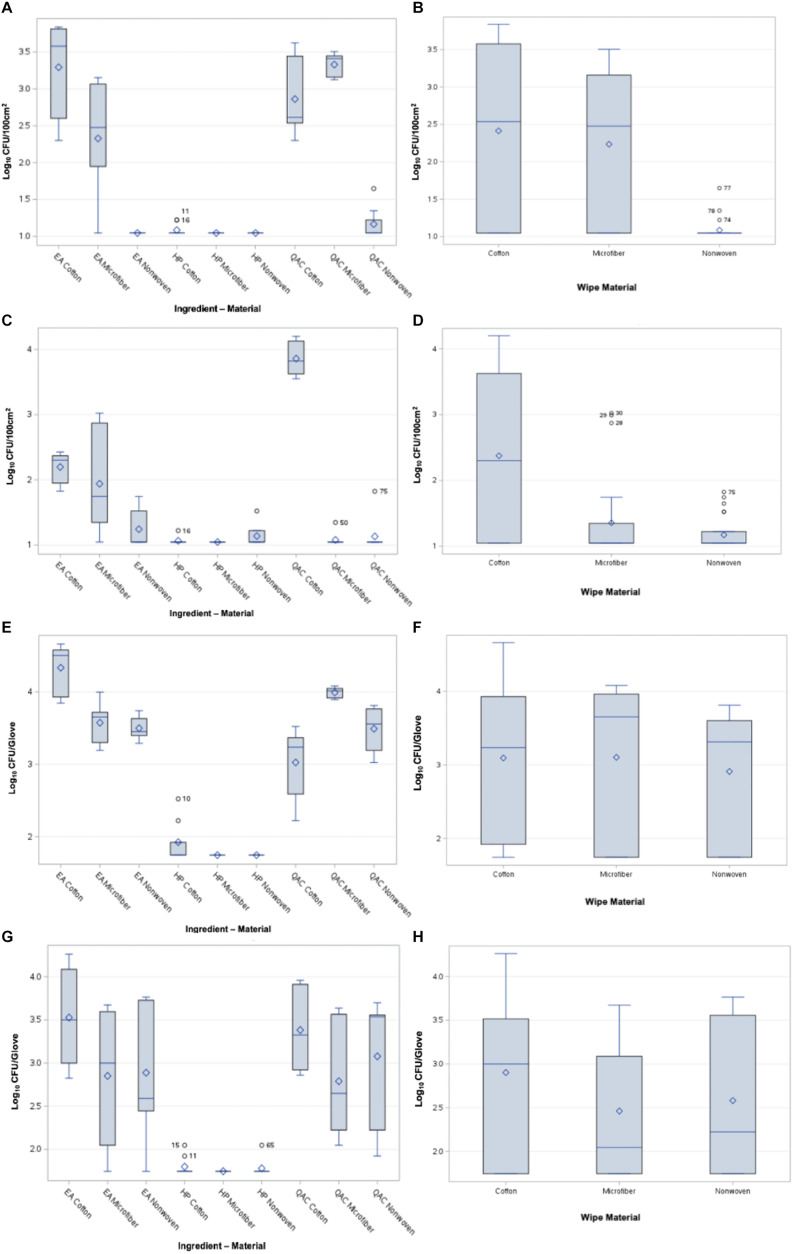
Panel **A** and **B**. Comparison of viable *S. aureus* transferred from the wipe to the board for each active ingredient and material type combination. One layer of wiping material was present between the inoculated wipe and Formica surface. Panel **C** and **D**. Comparison of viable *P. aeruginosa* transferred from the wipe to the board. Panel **E** and **F**. Comparison of viable *S. aureus* transferred from the wipe to the glove. Glove was placed directly on the inoculated wiping material. Panel **G** and **H**. Comparison of viable *P. aeruginosa* transferred from the wipe to the glove. Glove was placed directly on the inoculated wiping material.

The transfer of bacteria through an uncontaminated wipe layer to Formica surfaces was significantly impacted by both wiping material and liquid active ingredient (*P*<.0001; Figures 3A,D). HP-impregnated wipes consistently transferred the lowest bacterial counts (*P*<.05; Figures 3A,C). Microfiber wipes inoculated with *P. aeruginosa* transferred 1.4 ± 0.6 log_10_ CFU/100 cm,^2^ compared to cotton wipes which transferred 2.4 ± 1.2 log_10_ CFU/100 cm^2^ (*P*<.05; Figure 3D). No significant difference was observed between microfiber and polypropylene wipes for *P. aeruginosa* (*P*>.05; Figure 3D). For *S. aureus*, cotton and microfiber wipes transferred significantly more bacteria than polypropylene wipes (*P*<.05; Figure 3B).

### Reduced wiping layers increased glove cross-contamination

When gloves were placed directly on inoculated wipes, bacterial transfer was significantly affected by both wiping material (*P*<.0149) and liquid active ingredient for both organisms (*P*<.0001; Figures 3E,H). For *S. aureus*, QAC and EA wipes transferred 3.5 ± 0.5 and 3.8 ± 0.5 log_10_ CFU/glove, respectively, compared to 1.8 ± .2 log_10_ CFU/glove for HP wipes (*P*<.05; Figure 3E). For *P. aeruginosa*, EA and QAC wipes transferred 3.1 ± 0.8 and 3.1 ± 0.7 log_10_ CFU/glove, respectively, compared to 1.8 ± 0.1 log_10_ CFU/glove for HP wipes (*P*<.05; Figure 3G).

For *S. aureus*, cotton and microfiber wipes each transferred 3.1 ± 1.1 log_10_ CFU/glove, while polypropylene wipes transferred 2.9 ± 0.9 log_10_ CFU/glove (Figure 3F). For *P. aeruginosa*, cotton wipes transferred 2.9 ± 0.9 log_10_ CFU/glove, significantly more than microfiber (2.5 ± 0.8 log_10_ CFU/glove) and polypropylene (2.6 ± 0.9 log_10_ CFU/glove) wipes (*P*<.05; Figure 3H).

## Discussion

Ingredient chemistry had a clear impact on the transfer of bacteria from contaminated wipes. HP-impregnated wipes reduced counts below detection and transferred the fewest bacteria, consistent with HP’s rapid, broad antimicrobial activity across different materials.^21,22^ QAC sanitizers transferred more bacteria with cotton and microfiber due to electrostatic binding of cationic QAC molecules to the negatively charged surfaces of cellulose and polyester/polyamide fibers, reducing the concentration of free active ingredient in solution.^10,16,23^ Polypropylene’s neutral, hydrophobic surface reduced this binding, allowing greater retention of QAC activity and lower cross-contamination. EA chemistry also allowed high bacterial transfer, consistent with its weak bacteriostatic activity requiring higher concentrations for inhibition.^24^ These differences between QAC- and EA-based formulations highlight the importance of selecting chemistries with rapid bactericidal activity rather than relying on cleaning or physical removal alone.

Material type also significantly impacted bacterial transfer, which appears to be driven by interactions between the chemical formulation and the physical properties of the wipe substrate. Polypropylene wipes had the lowest cross-contamination risk compared to cotton and microfiber, consistent with prior findings on fiber structure and surface chemistry differences affecting disinfectant-substrate compatibility.^11,16,19^ The liquid volumes applied to each material (15 mL for polypropylene vs 121 mL for microfiber and 146 mL for cotton) were based on each material’s absorptive capacity to achieve full saturation, reflecting real-world conditions where prewetted wipes are manufactured at their saturation point.^10,17,18^ These volume differences are a potential confounder, as the total mass of active ingredient delivered to cotton and microfiber was proportionally greater than to polypropylene. However, cotton and microfiber wipes still transferred more bacteria despite receiving more disinfectant, which suggests that material-chemistry interactions—particularly QAC binding to charged fiber surfaces—had a greater influence on transfer than the volume of disinfectant applied.^16,23^ Future studies should evaluate the relationship between disinfectant volume, saturation level, and bactericidal efficacy across wipe materials to better characterize this interaction.

Microfiber wipes showed variable performance depending on layering and organism. Microfiber’s fine, asterisk-shaped fibers promote wicking, but multiple layers reduce this effect, trapping bacteria in the substrate and limiting transfer regardless of chemistry.^19^ Directly placing contaminated wipes on surfaces or gloves resulted in higher transfer, particularly when using QAC and EA formulations. As layers increased, transfer to both surfaces and gloves decreased across materials, indicating that wipe layering is an important factor in cross-contamination risk independent of chemistry. Even a single additional layer between the inoculated surface and the glove reduced bacterial transfer. These results highlight the combined role of chemistry selection, wipe material, and wipe-handling practices in reducing cross-contamination during cleaning.

## Limitations

A key limitation was the difference in inoculation placement between models (Figures 1B,C). In experiment 1, bacteria were applied to the bottom exterior of the wipe, while in experiment 2 they were inoculated on the interior before folding. This may have allowed migration toward both the surface and glove, limiting direct comparison of transfer across wipe layers. Another limitation was the substantially different disinfectant volumes required to saturate the three wipe materials. Because cotton and microfiber wipes were larger and require higher liquid uptake than polypropylene to reach saturation, the delivered volume of active ingredient differed across materials. Although these volumes were based on each material’s absorptive capacity and reflect conditions of actual use, they cannot be fully separated from the effects of wipe substrate or chemistry on bacterial transfer. Whether lower volumes maintain efficacy on cotton and microfiber or higher volumes improve polypropylene performance remains unclear. Future studies should determine the minimum effective volume for each material and systematically evaluate how liquid volume, wipe type, and chemistry interact to influence bactericidal efficacy and cross-contamination.

## Conclusion

This study underscores the importance of selecting appropriate chemistry and wiping materials to prevent cross-contamination during cleaning and disinfection. Meltblown polypropylene was the most effective material at minimizing cross-contamination. When multiple layers of wiping material were used, microfiber wipes showed a reduced risk of cross-contaminating bacteria, particularly against *P. aeruginosa*. Transfer of bacteria to uncontaminated surfaces was observed for most QAC sanitizer and EA cleaner combinations. Overall, polypropylene wipes combined with a hydrogen peroxide-based disinfectant solution provide the most reliable and effective option for minimizing microbial spread, offering a practical strategy for improving hygiene and infection control in healthcare and other high-risk environments.
